# Increased circulating uric acid aggravates heart failure via impaired fatty acid metabolism

**DOI:** 10.1186/s12967-023-04050-5

**Published:** 2023-03-16

**Authors:** Bowen Lou, Haoyu Wu, Hannes Ott, Katrin Bennewitz, Chen Wang, Gernot Poschet, Hui Liu, Zuyi Yuan, Jens Kroll, Jianqing She

**Affiliations:** 1 Cardiovascular Department, The First Affiliated Hospital of Xi’an Jiaotong University, Xi’an, 710061 Shaanxi China; 2 Key Laboratory of Environment and Genes Related to Diseases, Ministry of Education, Xi’an, 710061 Shaanxi China; 3 Department of Vascular Biology and Tumor Angiogenesis, European Center for Angioscience (ECAS), Medical Faculty Mannheim, Heidelberg University, Mannheim, Germany; 4 Metabolomics Core Technology Platform, Centre for Organismal Studies, Heidelberg University, Heidelberg, Germany; 5 First Affiliated Hospital of Xi’an Jiaotong University, Xi’an, 710061 Shaanxi China

**Keywords:** Uric acid, Fatty acid, Sterol regulatory element binding proteins 1, Heart failure

## Abstract

**Background:**

Increased circulating uric acid (UA) concentration may disrupt cardiac function in heart failure patients, but the specific mechanism remains unclear. Here, we postulate that hyperuremia induces sterol regulatory element binding protein 1 (SREBP1), which in turn activate hepatic fatty acid biosynthesis response, leading to cardiac dysfunction.

**Methods and results:**

Increased circulating uric acid was observed in heart failure patients and inversely correlated to cardiac function. Besides, uric acid correlated to circulating lipids profile based on metabolomics in heart failure patients. Using cultured human hepatoellular carcinomas (HepG2) and Tg(myl7:egfp) zebrafish, we demonstrated that UA regulated fatty acid synthase (FASN) via SREBP1 signaling pathway, leading to FFA accumulation and impaired energy metabolism, which could be rescued via SREBP1 knockdown. In ISO treated zebrafish, UA aggravated heart failure via increased cardiovascular cavity size, decreased heart beats, pericardial edema and long-stretched heart deformation.

**Conclusions:**

Our findings suggest that UA-SREBP1-FASN signaling exacerbates cardiac dysfunction during FFA accumulation. Identification of this mechanism may help in treatment and prevention of heart failure.

**Supplementary Information:**

The online version contains supplementary material available at 10.1186/s12967-023-04050-5.

## Introduction

Serum uric acid (SUA) is the end product of both dietary and endogenous purine metabolism [1]. In clinical practice, hyperuricemia is common in patients with chronic HF patients, and elevated UA levels have been independently associated with adverse long-term outcomes [2]. A meta-analysis of 18 studies with over 33,000 sample sizes suggest SUA is positively associated with the risk of all-cause mortality, cardiovascular death and combined death or cardiac events in CHF patients [3]. After multivariable analyses, UA was confirmed to be significantly associated with heart failure (HF) and cardiogenic shock in acute coronary syndromes (ACS) patients [4]. Arslan and teams found UA combined with NT-proBNP seemed to be a stronger predictor for all-cause mortality and HF hospitalization [5]. However, through the pooled analysis of six studies, Xu and colleagues demonstrated uric acid-lowering therapies did not improve ejection fraction (EF), N-terminal pro hormone B-type natriuretic peptide (NT-proBNP), as well as all-cause mortality and cardiovascular death (CVD) in HF patients, considering UA may just be a risk marker of HF [6]. Although a plethora of evidence supports the association between hyperuricemia and increased mortality risk, it remains quite ambiguous whether UA plays a role as a risk marker or risk factor in CVD and cardiometabolic disease.

Interestingly, several studies reported that elevated UA levels regulate the oxidative stress, inflammation and enzymes associated with free fatty acids (FFA) metabolism [7–9]. FFA are a byproduct of lipolysis that provide 60–70% of the adenosine triphosphate required by myocardial metabolism under physiological condition [10], but may also be associated with HF when FFAs are altered relative to levels under normal states [11]. A positive association between plasma FFA and incident HF was observed in the prospective cohort from Cardiovascular Health Study [12]. Additionally, spillover of FFA may damage the myocardium through increased β-oxidation and toxic lipid species accretion in the myocardium and present exacerbation of lipotoxicity [13]. Higher FFA levels are considered to be associated with endothelial dysfunction [14], cardiac inflammation and myocardial glucose metabolism inhibition [15], ischemic myocardial damage and arrhythmia [16, 17]. Nevertheless, the molecular mechanism regarding high UA induced impairment of lipid metabolic homeostasis and its potential “role” during the progression of HF remains incompletely understood.

Accordingly, the aims of this study were (i) to assess whether SUA levels would be elevated in HF patients compared with control subjects; (ii) to evaluate the association between SUA, FFA levels and cardiac remodelling and dysfunction; (iii) to determine whether SUA would regulate FFA in vitro and vivo; and (iv) to elucidate the molecular mechanism regarding SUA induced lipid metabolic impairment during HF progression.

## Materials and methods

### Study approval

All patients had been recruited from the First Affiliated Hospital of Xi’an Jiaotong University. Written informed consent was obtained from all study participants, with ethnic committee approval at the First Affiliated Hospital of Xi’an Jiaotong University and was conducted according to the Declaration of Helsinki.

All experimental procedures on animals were approved by Medical Faculty Mannheim (license no: I-19/02) and carried out in accordance with the guidelines from Directive 2010/63/EU of the European Parliament on the protection of animals used for scientific purposes.

### Zebrafish husbandry and zebrafish lines

Zebrafish lines, *Tg*(*fli1:EGFP*) [18] and *Tg*(*myl7:egfp*) [19], were raised and staged as described [20] under standard husbandry environment. Embryos/larvae were kept in E3 media at 28.5 °C with/without PTU (2.5 mL in 25 mL) to suppress pigmentation. Adult zebrafish were kept under 13 h light/11 h dark cycle and fed with living shrimps in the morning and fish flake food in the afternoon.

### Microscopy and analysis of heart alterations in zebrafish larvae

For in vivo imaging of the zebrafish heart structure, *Tg*(*myl7:egfp*) larvae were anaesthetized in 0.0003% tricaine at 72 hpf, and mounted head up in 0.8% low-melt agarose and placed on a coverslip bottom dish in wells made from a layer of 3% agarose. Images were collected using a Zeiss LSM510 confocal microscope equipped with a 40× water objective. In each stack, 60 confocal slices spanning the expression of Tg(myl7:egfp) were collected at 1–1.5 µm intervals and merged by ImageJ.

### Pharmacological treatment of zebrafish embryos/larvae

Fertilized zebrafish embryos were transferred into 6-well plate, around 30 embryos per well with 5 mL eggwater. At 24 hpf the chorion of zebrafish embryos was removed using sharp forceps and 0.003% PTU was added to the eggwater. Uric Acid (U2625; Sigma-Aldrich), Isopropenrol, Hydrochloride (420355; Sigma-Aldrich), Palmitic Acid (P0500; Sigma-Aldrich) and Stearic Acid (S4751; Sigma-Aldrich) treatments were started at 3 hpf and continued until the end; Medium was changed every day.

### Cell cultures and treatments

Human hepatocellular carcinoma cells (HepG2) were cultured in DMEM containing 10% fetal bovine serum, 100 U/mL penicillin and streptomycin at 37 °C in 95% air and 5% CO2. HepG2 cells at 50–70% confluence were transfected with 40 nM SREBP1 siRNA or scramble control siRNA using Lipofectamine 2000 (Invitrogen) for 24 h, and then treated with 1 mM uric acid (Sigma-Aldrich) for another 48 h. Human SREBP1 siRNA and scrambled control siRNA were purchased from Santa Cruz Biotechnology.

### Reverse-transcription quantitative polymerase chain reaction analysis (RT-qPCR)

Total RNA was isolated from *TG*(*fli1:EGFP*) zebrafish larvae at different time points using the RNeasy Mini Kit following the manufacturer’s protocol (Qiagen). First-strand cDNA was generated from 1 μg RNA using the Maxima First Strand cDNA Synthesis Kit according to the manufacturer’s protocol (Thermo Scientific). Primer design for zebrafish was done using NCBI or by Roche Universal Probe Library Assay Design Center and primers are listed in Additional file 1: Table S1. All samples were used with Power SYBR™ Green PCR Master Mix Kit in 96-well reaction plates. The qPCR reaction was performed with QuantStudio 3 Real-Time-PCR-System.

For cell culture, RNA was isolated from cultured cells by using TRIzol (Invitrogen). The mRNA level was measured by using SYBR Green (Bio-Rad) with β-actin–actin as internal control. Primer sequences used for qPCR are in Additional file 1: Table S1.

### Metabolomic analysis

For zebrafish, detection was done in cooperation with the Metabolomics Core Technology Platform from the Centre of Organismal Studies Heidelberg. At 96 hpf, around 50 zebrafish larvae per measurement were anaesthetized with 0.003% tricaine and snap frozen. Adenosine compounds, thiols, free amino acids, fatty acids and primary metabolites were measured as previously described [21].

For cell culture and human serum FFA measurement, targeted metabolomics were applied for FFA detection. The sample preparation, instrumentation, metabolic annotation and data analysis procedure are referred in our previously published methods with minor modification [22–28].

### Western blot analysis

For Western blot analysis, HepG2 cells were lysed in the RIPA buffer supplemented with protease inhibitors (Thermo Scientific). Lysates were separated by SDS-PAGE and immunoblotted with antibodies as indicated. Western blot analysis was performed with the following antibodies: SERBP1 (Abcam, ab28481), Fatty acid synthase (Cell Signaling, 3180S), β-actin (Santa Cruz Biotechnology, sc-47778).

### Clinical data collection

4772 consecutive patients admitted to the cardiology department of the First Affiliated Hospital of Xi’an Jiaotong University for AMI between January 2016 and December 2020 were enrolled. The inclusion criteria were confirmed admission diagnosis of metabolic syndrome. The exclusion criteria were: (1) severe nonmetabolic disease with an expected survival of less than 1 year and unwillingness to participate; (2) patients over the age of 80 years or living far away from the hospital’s catchment area. A patient could only be included once. The medical records of the patients were collected from the Biobank of the First Affiliated Hospital of Xi’an Jiaotong University, which contains the identified data derived from raw medical records, information about patients’ detailed medical histories, present medication, biochemical and echocardiography results. Written informed consent was obtained from all study participants, with ethnic committee approval at the First Affiliated Hospital of Xi’an Jiaotong University.

### Softwares and statistics

Experimental results are expressed as mean with standard deviation (mean ± SD). Statistical significance between different groups was analysed using Student’s *t*-test or one-way ANOVA (followed either by post hoc Bonferroni’s, Sidak’s multiple comparison). For clinical results, simple t-test was used to compare continuous variables after normality test and χ^2^ test was used to compare categorical variables. The simple linear analysis was used to calculate the correlation. GraphPad Prism 8.3.0 was used for analyses and* p* values of 0.05 were considered as significant: *p < 0.05, **p < 0.01, ***p < 0.001, ****p < 0.0001. Bioinformatic analysis was performed using the OmicStudio tools at https://www.omicstudio.cn/tool and MetaboAnalyst at https://www.metaboanalyst.ca/MetaboAnalyst/ModuleView.xhtml. Schematic diagram was performed via Figdraw and Powerpoint.

## Results

### UA was negatively correlated to cardiac function and positively correlated to circulating fatty acid level in HFrEF patients.

**Fig. 1:**
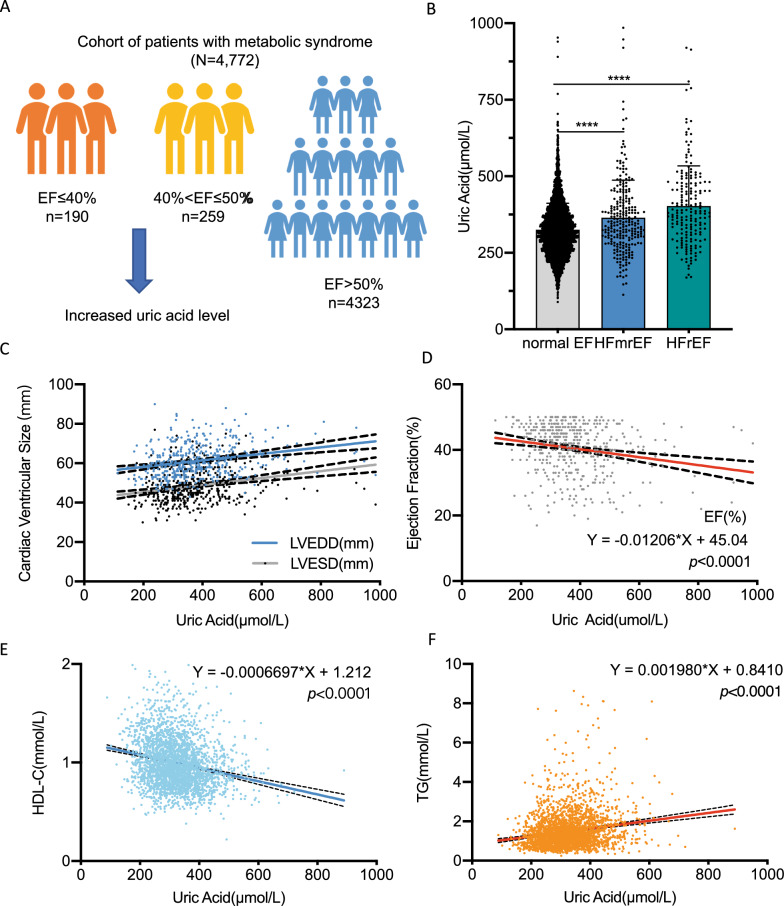
Circulating uric acid was negatively correlated to cardiac function, high density lipoprotein and positively correlated to triglycerides in metabolic syndrome cohort. **A** Schematic diagram of the correlation between uric acid level and EF value in metabolic syndrome: in the patients with EF ≤ 40% (n = 190) and 40% < EF ≤ 50% (n = 259), increased uric acid level was observed; **B** Column plot of uric acid level among HFrEF, HFmrEF and normal EF patients; **C**, **D** In metabolic syndrome cohort: circulating uric acid was significantly negatively correlated to cardiac function: positively correlated to cardiac ventricular size (**C**) and negatively correlated to EF value (**D**); **E** Circulating uric acid was negatively to high density lipoprotein; **F** Circulating uric acid was positively to triglycerides in metabolic syndrome patients. Each single dot represents one patient’s data in the above plots. EF, ejection fraction; HFmrEF, heart failure with mid-range ejection fraction; HFrEF, heart failure with reduced ejection fraction; HDL-C, high density lipoprotein; TG, triglycerides

To investigate the correlation between UA and cardiac function, we extracted clinical data of 4772 patients diagnosed with metabolic syndrome from First Affiliated Hospital of Xi’an Jiaotong University from Biobank, including 190 heart failure with reduced ejection fraction (HFrEF) patients, 259 heart failure with mid-range ejection fraction (HFmrEF) patients and 4323 normal ejection fraction (EF) patients. Baseline information were shown in Table 1. In general, the mean age was 63.76 ± 10.33 in the HFrEF group, 64.03 ± 11.25 in the HFmrEF group and 63.09 ± 10.67 in normal EF group. Female was account for 43.15%, 42.93% and 42.06% in the three sets respectively (Table 1). UA value was significantly higher in HFrEF and HFmrEF groups compared to normal EF group (Fig. 1a, b). Linear regression was performedand it is identified that UA negatively correlated to cardiac function, as quantified by cardiac ventricular size (Fig. 1c left ventricular end-diastolic dimension, LVEDD; left ventricular end-systolic dimension, LVESD) and EF value (Fig. 1d). Then, after adjusting for age, sex, blood glucose and lipid leveI (HbA1C and LDL-C), liver and kidney function (ALT, AST and Cre), multiple linear regression analyses revealed that UA was independently correlated with EF level (b =  − 0.026, β =  − 0.297, *p* = 0.043) (Additional file 1: Table S2). In addition, UA also significantly correlated to blood lipid profiles, with negative correlation to high density lipoprotein (HDL-C, Fig. 1e) and positively to TG (triglycerides, Fig. 1f).

**Table 1:** Baseline information of the metabolic syndrome cohort (big cohort)

	HrEF (n = 190)	HFmrEF (n = 259)	nornal EF (n = 4323)	*p* value
Age	63.76 ± 10.33	64.03 ± 11.25	63.09 ± 10.67	ns
Sex (Female%)	43.15	42.93	42.06	ns
EF (%)	33.20 ± 5.74	45.76 ± 2.77	67.09 ± 5.94	*p* < 0.001
LVEDD (mm)	65.56 ± 8.46	57.85 ± 6.19	49.23 ± 4.09	*p* < 0.001
LVESD (mm)	54.67 ± 8.31	46.05 ± 3.89	30.59 ± 3.96	*p* < 0.001
UA (umol/L)	402.87 ± 131.12	364.39 ± 123.03	325.08 ± 86.72	*p* < 0.001
AST (U/L)	30.56 ± 34.23	30.50 ± 34.90	25.42 ± 39.01	ns
ALT (U/L)	33.97 ± 48.55	36.88 ± 37.41	29.65 ± 27.44	ns
Cre (μmol/L)	97.12 ± 112.65	87.58 ± 73.19	66.53 ± 32.30	*p* < 0.001
Chol (mmol/L)	3.56 ± 0.88	3.60 ± 0.91	3.88 ± 1.10	ns
TG (mmol/L)	3.24 ± 1.15	3.43 ± 1.09	3.54 ± 1.39	ns
HDL-C (mmol/L)	0.93 ± 0.23	0.94 ± 0.21	1.06 ± 0.29	ns
LDL-C (mmol/L)	2.17 ± 0.90	2.17 ± 0.31	2.21 ± 0.87	ns
HbA1C (%)	6.40 ± 1.31	6.50 ± 1.48	6.04 ± 1.10	*p* < 0.001

EF, ejection fraction; LVEDD, left ventricular end diastolic diameter; LVESD, left ventricular end systolic diameter; UA, uric acid; ALT, alanine transaminase; AST, aspartate transaminase; Cre, creatinine; Chol, cholesterol; TG, triglycerides; HDL-C, high density lipoprotein; LDL-C, high density lipoprotein

**Fig. 2:**
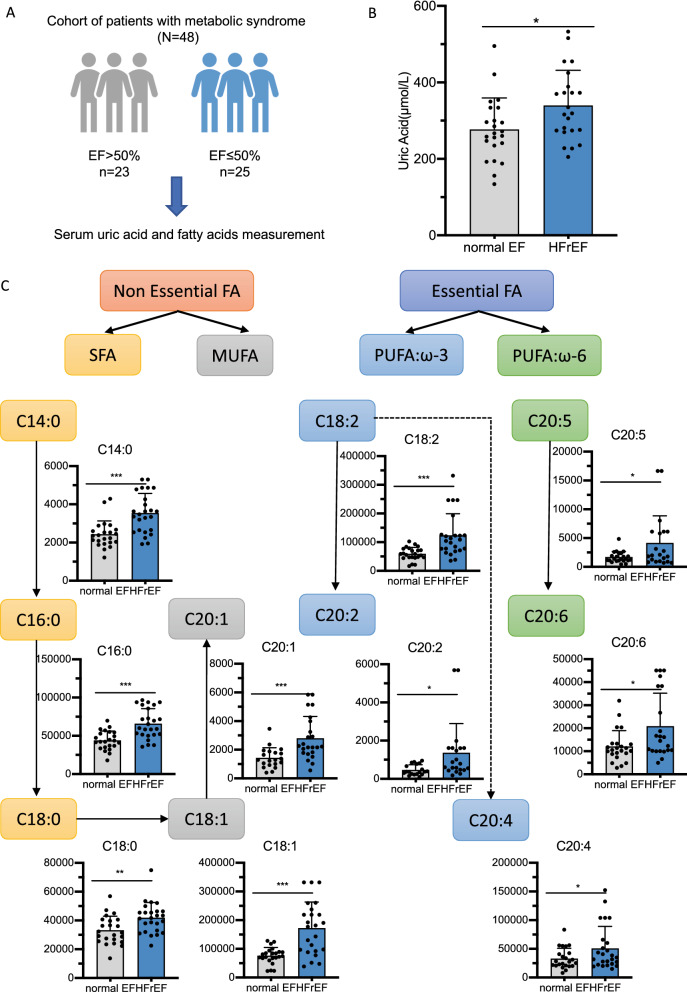
Increased FFA and uric acid levels in heart failure patients. **A** Schematic diagram of the metabolomics measurements in HFrEF and control patients with metabolic syndrome. **B** Column plot of uric acid level among HFrEF and normal EF patients in this cohort; **C** Column plot of each FFA level among HFrEF and normal EF patients in this cohort. N = 23–25 per group as the dot showed in the column plot. For statistical analysis student *T* test was applied, *p < 0.05, **p < 0.01, ***p < 0.001. UA, uric acid; HFrEF, heart failure with reduced ejection fraction; FFA, free fatty acid; SFA, saturated fatty acid; MUFA, monounsaturated fatty acid; PUFA, polyunsaturated fatty acid

**Fig. 3:**
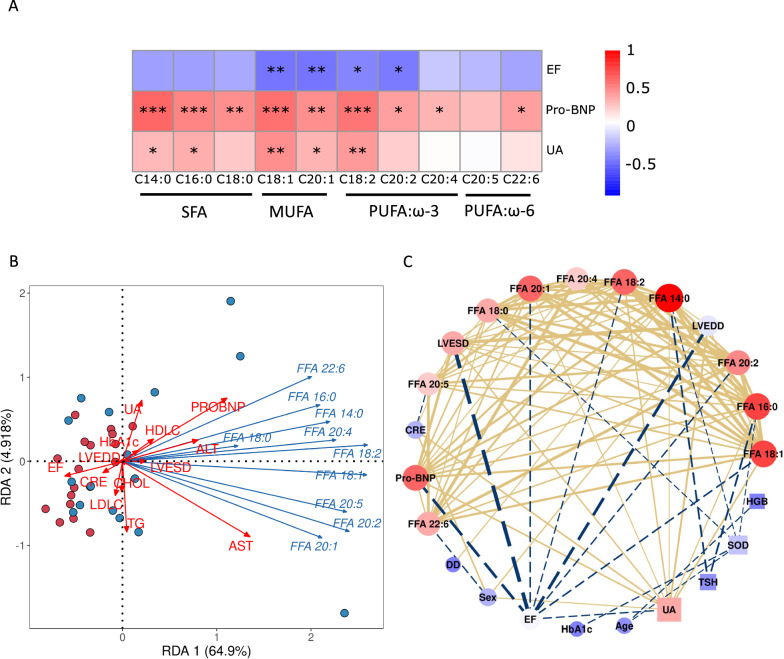
Uric acid correlated to circulating lipids profile based on metabolomics in heart failure patients. **A** Heatmap showed uric acid positively correlated and cardiac function reversely correlated to circulating lipids. The color in each cell represents value of correlation calculated by Spearman’s rank correlation coefficient. *p < 0.05, **p < 0.01, ***p < 0.001. **B**, **C** network analysis between fatty acid and clinical parameters. UA, uric acid; HFrEF, heart failure with reduced ejection fraction; FFA, free fatty acid; SFA, saturated fatty acid; MUFA, monounsaturated fatty acid; PUFA, polyunsaturated fatty acid

Since the data from clinical cohorts with metabolic syndrome revealed significant higher UA levels in patients with impaired cardia function, and possible correlation between UA and circulating lipids levels, we then investigated the relationship between UA and specific circulating lipid components. To this end, targeted metabolomics analysis of FFAs were applied in participants with normal EF and reduced EF (Fig. 2a); the basic characters for this cohort were shown in Additional file 1: Table S3. In this age and gender matched cohort, UA was around 20% higher in HFrEF patients as compared to controls (Fig. 2b 276.72 vs. 366.90 umol/L *p* < 0.001). Both non-essential and essential FA profiles were increased in heart failure patients (Fig. 2c). Almost all non-essential FFA and C18:2 showed positive correlation with UA concentration and reverse association with cardiac function in the whole cohort. Besides, in network correlation analysis, UA also showed “stronger positive connection” with FFA alteration as compared to other clinical biochemical parameters, including but not limited to: HbA1c, CRE, TSH and HGB (Fig. 3a–c, Additional file 1: Fig. S1).

### UA aggravates ISO induced heart failure and caused energy metabolism in zebrafish

**Fig. 4:**
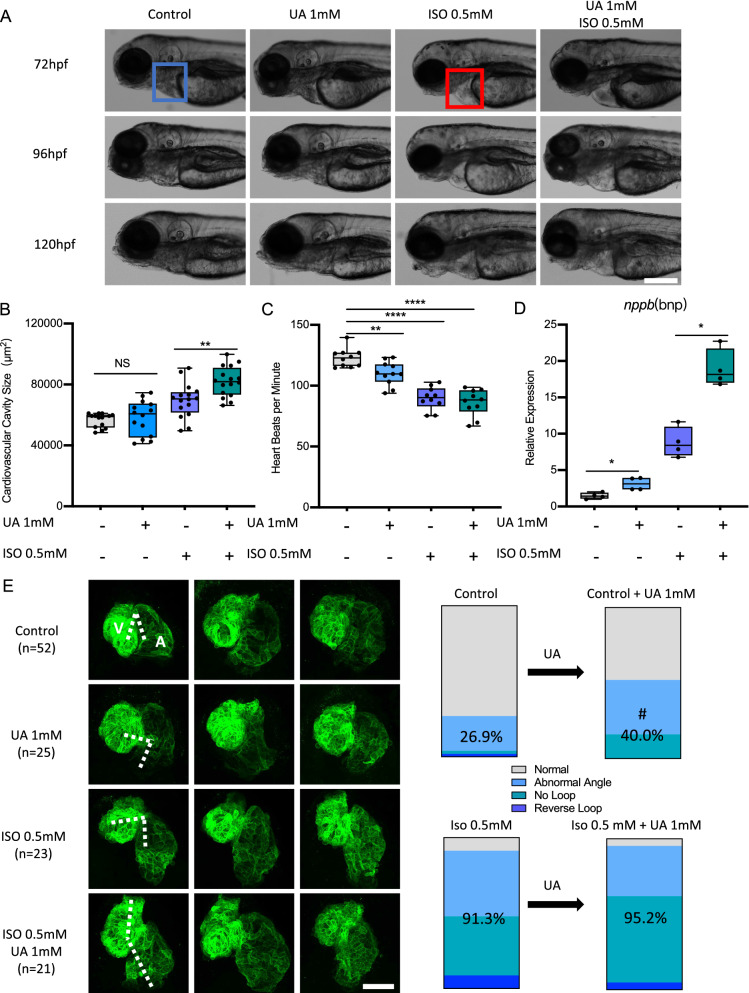
Uric acid aggravated ISO induced heart failure in zebrafish. **A**–**D** 1 mM Uric acid treatment aggravated 0.5 mM ISO induced heart failure in *Tg*(*fli1:EGFP*) zebrafish larvae at 72, 96 and 120 hpf. Representative light microscopic images **A** showed the gross morphology of zebrafish larvae, blue frame indicates normal cardiovascular cavity and red frame indicates edema cardiovascular cavity, white scale bar = 200 μm; Heart failure was quantified by increased cardiovascular cavity size (**B**), decreased heart beats per minute (**C**) and increased *nppb* expression (**D**) at 120hpf, expression of mRNA was analysed by RT-qPCR and was normalized to both *b-actin* and *b2m.* In each group, 10–16 zebrafish larvae were used in **A**–**C**, each single dot represent one zebrafish larva. In **D** one single dot represents one zebrafish cluster’s data, which includes 20–30 zebrafish larvae. For statistical analysis one-way ANOVA followed by Sidak’s multiple comparison test was applied, *p < 0.05, **p < 0.01, ****p < 0.0001. E.1 mM UA treatment increased long-stretched heart with loop failure, including abnormal angle, reverse loop and no loop phenotype as compared to controls in *Tg*(*myl7:EGFP*) zebrafish larvae at 72hpf. Also, 1 mM UA and ISO co-treatment aggravated “no loop” phenotype ratio as compared to ISO treatment. N = zebrafish larvae analysed in each group. Dash indicates angle between atrium and ventricle, white scale bar = 50 μm. A, atrium; V, ventricle. Percentage in the column represents “abnormal heart phenotype” ratio in each group, including abnormal angle, reverse loop and no loop. ^#^*p* < 0.05 as compared to controls. UA, uric acid; ISO, isoproterenol

To further understand the specific mechanism how UA regulated FFA levels and heart fuction, zebrafish experiments were performed, as zebrafish embryo can tolerate the absence of blood flow for few days because its oxygen is delivered by diffusion rather than by the cardiovascular system, making it an excellent model for studying heart failure [29]. 1 mM UA was used for the study to mimick the hyperuricemia conditions in patients. At first, 1 mM UA treatment can slightly decrease heart rate and increase natriuretic peptide B (*nppb/pro-BNP*) level, but no alteration in cardiovascular size compared to control larvae (Fig. 4a–d) occured; Then, isoproterenol (ISO), a β-adrenergic receptor agonist, commonly used to trigger cardiac hypertrophy in animal models and cardiomyocytes [30], was used to construct heart failure model in zebrafish. According to the previous study, 1 mM ISO can lead to severe heart failure in zebrafish larvae [31]. To understand the effect of UA on cardiac function in the present study, 0.5 mM Iso was used in the following experiments to mimic a moderate heart failure. 1 mM UA treatment aggravated 0.5 mM ISO induced moderate heart failure in *Tg*(*fli1:EGFP*) zebrafish larvae at 72, 96 and 120 hpf (Fig. 4a), quantified by around 100 percent increased cardiovascular cavity size (B), decreased heart beats per minute (C) and up to 20-fold increased *nppb* expression (D).

To investigate the exact cardiovascular structure alteration after UA treatment, *Tg*(*myl7:egfp*) zebrafish larvae were applied, in which cardiomyocytes were marked with green fluorescence protein [32]. 0.5 mM ISO induced zebrafish heart failure caused pericardial edema and long-stretched heart with increased angle between atrium and ventricle (looping failure) at 3 dpf (Fig. 4e). 1 mM UA treatment aggravated ISO induced heart failure by increasing “no loop” phenotype, from 39.1 to 57.1% (Fig. 3e). Furthermore, in metabolomics analysis, UA aggravated ISO induced heart failure and lead to impaired energy metabolism in zebrafish (Additional file 1: Fig. S2).

### UA aggravated heart failure is most likely driven by FFA accumulation through SREBP1/FASN pathway

**Fig. 5:**
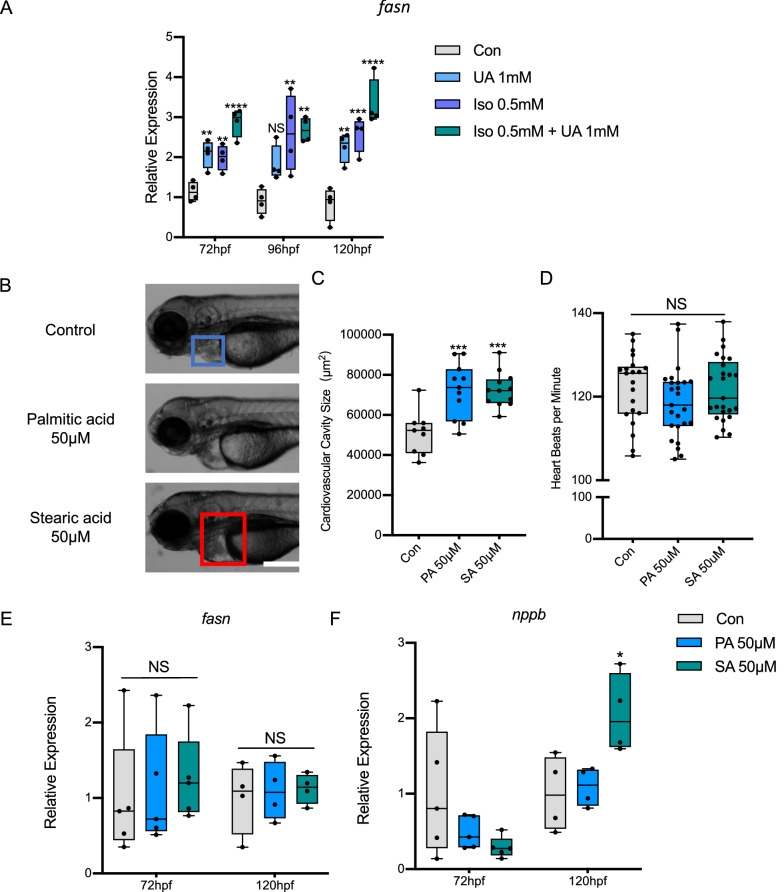
Uric acid aggravated heart failure is most likely driven by FFA accumulation through SREBP1/FASN pathway. **A** 1 mM UA treatment and 0.5 mM ISO induced increased *Fasn* and *Srebp1* mRNA expression in *Tg*(*fli1:EGFP*) zebrafish larvae at 72, 96 and 120 hpf. **B**–**F**, FFA treatments, including 50 μM Palmitic acid and Stearic acid incubation, caused heart failure in *Tg*(*fli1:EGFP*) zebrafish larvae, quantified by increased cardiovascular cavity size (**B**, **C**) and *nppb* level at 120hpf (**E**), heart beats per minute (**D**) and *Fasn* (**F**) level were not altered. In **A**, **E**, **F**, expression of mRNA was analysed by RT-qPCR and was normalized to both *b-actin* and *b2m*, each single dot represents one zebrafish cluster’s data, which includes 20–30 zebrafish larvae. In **B**–**D**, 9–25 zebrafish larvae were used in each group and each single dot represents one zebrafish’s data. For statistical analysis one-way ANOVA followed by Sidak’s multiple comparison test was applied, *p < 0.05, **p < 0.01, ***p < 0.001, ****p < 0.0001. UA, uric acid; ISO, isoproterenol; PA, palmitic acid; SA, stearic acid. FFA, free fatty acid

**Fig. 6:**
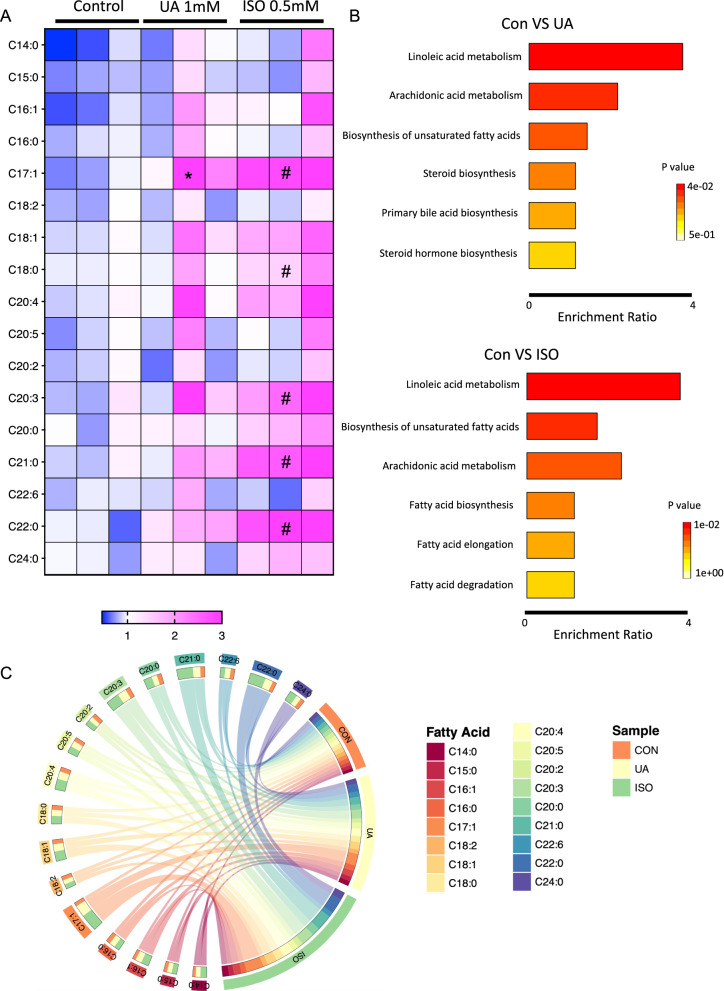
Uric acid and ISO induced heart failure leads to impaired fatty acid metabolism in zebrafish. **A** Heatmap of several increased fatty acids in UA and ISO induced heart failure zebrafish larvae at 96 hpf. The color in each cell represents the expression of each zebrafish cluster sample as the scale bar showed, each zebrafish cluster includes 50 zebrafish larvae. For statistical analysis, two-way analysis of variance (ANOVA) followed by Holm-Sidak test was used to evaluate the statistical significance of differences among three groups. *: Con versus UA *p* < 0.05; #: Con versus ISO *p* < 0.05; **B** Enrichment analysis of fatty acid profiles and TOP6 pathways between Con versus UA and Con versus ISO group. **C** Circos plot of fatty acids among each group

To identify fatty acid accymulation after UA aggravated heart failure, expression analyses of lipid metabolism related genes including fatty acid synthase (Fasn), fatty acid desaturase 2 (fads2), fatty acid elongase 2 (elov2), and stearoyl-CoA desaturase (scd) were performed (Additional file 1: Fig. S3), and Fasn was increased in UA treated zebrafish heart failure model (Fig. 5a), indicating the regulatory role of UA in heart failure via dysfunction of FFA synthesis. Lipid profile further identified increased fatty acids in both UA treated and ISO induced heart failure zebrafish larvae at 96 hpf (Fig. 6a, c). Through MetaboAnalyst analysis, enrichment of lipid metabolism pathways were identified in both groups as compared to controls, such as linoleic acid metabolism, biosynthesis of unsaturated fatty acids and arachidonic acid metabolism (Fig. 6b). At last, to further clarify the causal relationship between UA, fatty acid and heart failure, fatty aicd incubation experiments was performed in zebrafish larvae. Saturated fatty acids, including 50 uM palmitic acid and stearic acid, results in partially heart failure, quantified by increased cardiovascular cavity size (Fig. 5b–d) and around 100 percent increased *nppb* expression (Fig. 5f). Besides, *fasn* was not altered (Fig. 5e). This implied that external fatty acids can directly contribute to heart failure; whereas, UA may induce heart failure possibly through pathological FFA regulation.

**Fig. 7:**
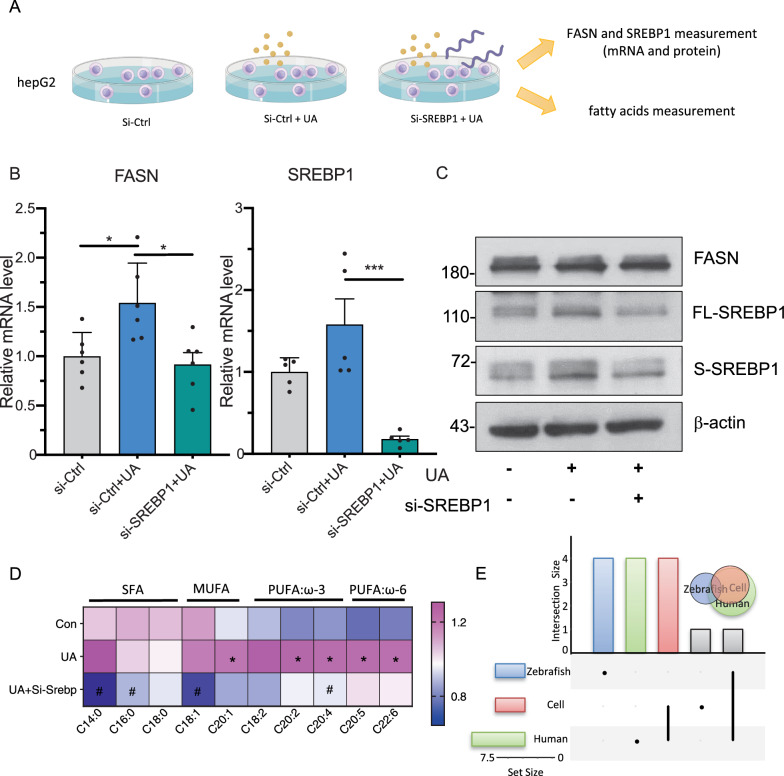
Uric acid treatment increases FASN/SREBP1 expression and FFA accumulation in HepG2 and can be rescued via SREBP1 knock down. **A** Schematic diagram of the cell culture, HepG2 were transfected with SREBP1 siRNA or control siRNA for 24 h followed by 1 mM UA treatment for another 48 h. The SERBP1 and FASN mRNA and protein levels, and fatty acids were measured and analyzed then. **B** Column plot of SERBP1 and FASN mRNA expression. N = 6 per each group. The average values of Si-Ctrl were standardized to 1, *p < 0.05 as compared to control group; For statistical analysis one-way ANOVA followed by Sidak’s multiple comparison test was applied, ^#^*p* < 0.05 as compared to UA group. **C** Western blot of SERBP1 and FASN protein expression, n = 3 per group. SREBP1 signaling can be detected as an ~ 68 splicing and 120 kDa full-length protein bands. **D** Heatmap showed uric acid positively correlated to several lipids in HepG2 cell and can be rescued via SREBP1 knock down. The color in each cell represents value of correlation calculated by Spearman’s rank correlation coefficient. *p < 0.05 as compared to control group; ^#^*p* < 0.05 as compared to UA group. **E** Venn diagram displays the distribution of shared FFA alteration after uric acid treatment in cell, zebrafish and human dataset. FASN, fatty acid synthase; SREBP1, sterol regulatory element binding protein 1

**Fig. 8:**
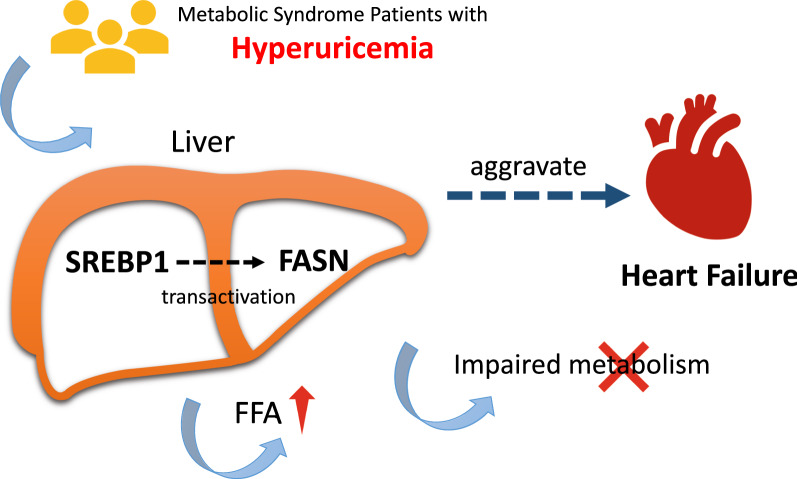
Graphical abstract/Mechanism plot. Increased UA level may regulate FASN via SREBP1 signaling pathway, leading FFA accumulation and impaired energy metabolism, which aggravates heart failure in metabolic syndrome patients ulteriorly

To validate the above findings revealed by zebrafish work and sought for potential signalling pathway in UA related heart failure, we performed UA incubation experiments in human hepatoellular carcinomas (HepG2), and transfected it with sterol regulatory element binding protein 1 (SREBP1) siRNA to check the rescue effects (Fig. 7a). First, UA increased around 50% mRNA expression of FASN and SREBP1, which were restored with siRNA induced SERBP1 knockdown (Fig. 7b). Also, we observed similar trend in protein level, with both increased FASN and full length SREBP1 (FL-SREBP1) and splicing SREBP1 (S-SREBP1) after UA treatment and rescued after SREBP1 silence (Fig. 7c). Finally, through FFA profile analysis, UA incubation was found to increase C20:0, C20:2, C22:6, etc., which was reversed after SREBP1 knock down (Fig. 7d), in accordance with our cell culture and clinical data (Fig. 7e), through our zebrafish, cell culture and clinical collection work, our findings suggest that UA-SREBP1-FASN signaling exacerbates cardiac dysfunction during FFA accumulation (Fig. 8).

## Discussion

In the present study, we have identified that UA correlates to FFA levels and cardiac function among patients with metabolic syndrome. UA correlated to FFA levels in HF patients and zebrafish model. High UA lead to impaired fatty acid metabolism and aggravate the lipotoxic heart injury. In addition, we demonstrate that targeting the UA-SREBP1-FASN pathway could be a possible therapeutic approach for HF. Our study provides new insights into heart failure with potentially important therapeutic implications.

The influence of serum uric acid level on cardiovascular disease is still controversial. Previous studies have explored the effect of uric acid on cardiovascular disease, but the results are inconsistent, and there are bias and other confounding factors [33, 34]. Our clinical exploration implies that there is an interaction between uric acid and fatty acid in the progression of heart failure. The zebrafish data further indicate that while fatty acid directly exagerrate heart failure, UA induce heart failure possibly through FFA regulation. This correlates with previous findings that UA itself is not an independent risk factor for HF, and UA-lowering medication could not ameliorate the longterm outcomes of HF.

It has previously been reported that patients with metabolic syndrome are prone to suffer from HF [35, 36]. Of note, hyperuricemia is closely related to metabolic syndrome. In human, it has been investigated that uric acid levels change with age and are closely related to obesity and insulin resistance [37, 38]. Hyperuricemia is often accompanied by inflammatory cytokines, elevated levels of oxidative stress, leading to the progression of heart failure [39]. On the other hand, ATP depletion caused by cardiac insufficiency could lead to adenine degradation into inosine, xanthine, hypoxanthine and uric acid, further increasing the level of circulating uric acid and resulting in a vicious cycle [40]. Although previous studies have confirmed the correlation between uric acid and heart failure, the key mechanisms and targets are still lacking. Our present study have provided evidence that UA could accelerating the progression of heart failure, at least in part through regulating FFA metabolism.

Metabolomics approach has enabled us to observe metabolic changes of specific disease from a more mechanistic view. In the present study, by exploring the FFA profile in HF patients and UA treated zebrafish and hepatic cells, we further clarify the change of fatty acid metabolism spectrum induced by uric acid treatment. In HF patients, we have observed increased circulating FFA levels including SFA, MUFA and PUFA. Correlation analysis has identified a positive correlation between UA levels and FFA, and a negative correlation between FFA and cardiac function as evaluated by EF and proBNP. In zebrafish, it is further clarified that UA itself could not causes HF but could aggrevate HF, accompanied by dyregulated FFA metabolism pathways. At last, it is testified in vitro that UA result in FFA increase in HepG2 cell line, which could be contereacted by SREBP1 silencing. The above result further establish the interaction of UA and FFA during HF progression.

The major novelty of the present study is that we point out that targeting UA-SREBP1-FASN axis might be a therapeutic approach for HF. SREBP1 has previously been identified as an intracellular cholesterol sensors located in the endoplasmic reticulum that provide feedback regulation of intracellular cholesterol. Recently it has been reported that UA could cause endothelial dysfunction via SREBP2 transactivation of YAP [41]. But it remains unclear whether UA regulate the SREBP1 pathway and cause dysregulated FFA metabolism. Our study further proved the regulatory function of UA in SREBP1 pathway. In addition, we have found that by downregulating SREBP1, UA could impair lipids metabolism homeostasis and aggravation of HF. This indicate that targeting SREBP1 could be a therapeutic approach for HF, especially metabolism syndrome related HF.

Our study has several limitations. Firstly, the regulatory function of SREBP1 has not been tested in vitro, further validation studies of SREBP1 KO animal model are needed. Secondy, the function of SREBP1 has only been tested in HepG2 cell line, bot not in human liver sample. Third, the correlation study could not explain why UA is increased in HF patients, although the mechanism may involve many factors, and needs to be further explored. Last, due to a retrospective study, several clinical parameters such as diuretic related therapy were not provided. As diuretic treatment (especially thiazides) was tightly associated with hyperuricemia, it would be quite interesting to investigate in HF patients in the future.

To sum, our findings suggest that UA-SREBP1-FASN signaling exacerbates cardiac dysfunction during FFA accumulation. Identification of this mechanism may help in treatment and prevention of heart failure.

## Supplementary Information

**Additional file 1. Fig. S1**: Correlations between FFA levels and clinical parameters in HF and control patients. Network analysis between fatty acid and clinical parameters. The color in each cell represents value of correlation calculated by Spearman‘s rank correlation coefficient. *p<0.05, **p<0.01, ***p<0.001. **Fig. S2**: UA aggravates ISO induced heart failure leads to impaired energy metabolism in zebrafish. **A** Heatmap of several metabolites in UA, ISO and co-treatment induced heart failure zebrafish larvae at 96 hpf. The color in each cell represents the expression of each zebrafish cluster sample as the scale bar showed, each zebrafish cluster includes 50 zebrafish larvae. **B** PCA analysis of every sample among each group. **C** VIP scores of the top metabolites among each group. **Fig. S3**: Expression analyses of elov2, scd and fads2 in zebrafish after UA and ISO treatment. 1 mM UA treatment and 0.5 mM ISO induced elov2, scd and fads2 mRNA expression in *Tg*(*fli1:EGFP*) zebrafish larvae at 72, 96 and 120 hpf. Expression of mRNA was analysed by RT-qPCR and was normalized to both *b-actin* and *b2m*, each single dot represents one zebrafish cluster’s data, which includes 20–30 zebrafish larvae. For statistical analysis one-way ANOVA followed by Sidak’s multiple comparison test was applied, *p < 0.05, **p < 0.01, ***p<0.001, ****p < 0.0001. Fads2, fatty acid desaturase 2; elov2. fatty acid elongase 2; scd, stearoyl-CoA desaturase. **Fig. S4**: Heatmap of fatty acids in HepG2 after UA treatment and Srebp1 knockdown. Heatmap of fatty acids in HepG2 after UA treatment and Srebp1 knockdown. The color in each cell represents expression of each cell culture sample as the scale bar showed, n = 3–4 per group. **Table S1**: Primers. **Table S2**: Multiple linear regression analysis in the metabolic syndrome cohort (big cohort). Dependent variable: EF value. UA, uric acid; ALT, alanine transaminase; AST, aspartate transaminase; Cre, creatinine; LDL-C, high density lipoprotein; *p < 0.05. **Table S3**: Baseline information of the heart failure cohort (small cohort for metabolomics detection). EF, ejection fraction; LVEDD, left ventricular end diastolic diameter; LVESD, left ventricular end systolic diameter; UA, uric acid; ALT, alanine transaminase; AST, aspartate transaminase; Cre, creatinine; Chol, cholesterol; TG, triglycerides; HDL-C, high density lipoprotein; LDL-C, high density lipoprotein.

## Data Availability

The datasets used or analysed during the current study are available from the corresponding authors on reasonable request.

## Abbreviations

EF: Ejection fraction
FASN: Fatty acid synthase
FFA: Free fatty acids
HepG2: Human hepatoellular carcinomas
HF: Heart failure
ISO: Isoproterenol
SREBP1: Sterol regulatory element binding protein 1
SUA: Serum uric acid
