# Supplementary material for: Increased circulating uric acid aggravates heart failure via impaired fatty acid metabolism
Source: J Transl Med. 2023 Mar 16;21:199. doi: 10.1186/s12967-023-04050-5 (PMC10018852; doi:10.1186/s12967-023-04050-5)
Supplement: Supplementary file 1 — Additional file 1. Fig. S1: Correlations between FFA levels and clinical parameters in HF and control patients. Network analysis between fatty acid and clinical parameters. The color in each cell represents value of correlation calculated by Spearman‘s rank correlation coefficient. *p<0.05, **p<0.01, ***p<0.001. Fig. S2: UA aggravates ISO induced heart failure leads to impaired energy metabolism in zebrafish. A Heatmap of several metabolites in UA, ISO and co-treatment induced heart failure zebrafish larvae at 96 hpf. The color in each cell represents the expression of each zebrafish cluster sample as the scale bar showed, each zebrafish cluster includes 50 zebrafish larvae. B PCA analysis of every sample among each group. C VIP scores of the top metabolites among each group. Fig. S3: Expression analyses of elov2, scd and fads2 in zebrafish after UA and ISO treatment. 1 mM UA treatment and 0.5 mM ISO induced elov2, scd and fads2 mRNA expression in Tg(fli1:EGFP) zebrafish larvae at 72, 96 and 120 hpf. Expression of mRNA was analysed by RT-qPCR and was normalized to both b-actin and b2m, each single dot represents one zebrafish cluster’s data, which includes 20–30 zebrafish larvae. For statistical analysis one-way ANOVA followed by Sidak’s multiple comparison test was applied, *p < 0.05, **p < 0.01, ***p<0.001, ****p < 0.0001. Fads2, fatty acid desaturase 2; elov2. fatty acid elongase 2; scd, stearoyl-CoA desaturase. Fig. S4: Heatmap of fatty acids in HepG2 after UA treatment and Srebp1 knockdown. Heatmap of fatty acids in HepG2 after UA treatment and Srebp1 knockdown. The color in each cell represents expression of each cell culture sample as the scale bar showed, n = 3–4 per group. Table S1: Primers. Table S2: Multiple linear regression analysis in the metabolic syndrome cohort (big cohort). Dependent variable: EF value. UA, uric acid; ALT, alanine transaminase; AST, aspartate transaminase; Cre, creatinine; LDL-C, high density lipoprotein; *p < 0.05. Table S3: Basel [file 12967_2023_4050_MOESM1_ESM.pdf]

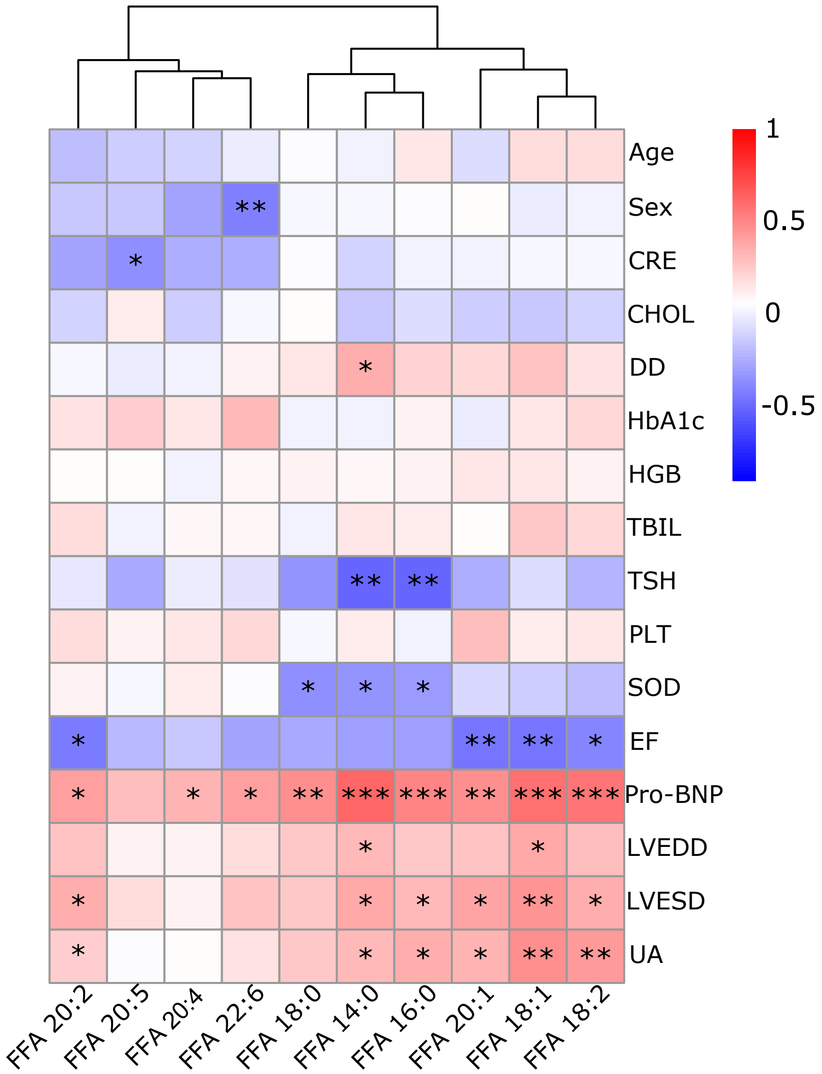

Figure S1.Correlations between FFA levels and clinical parameters in HF and control patients.

Network analysis between fatty acid and clinical parameters. The color in each cell represents value of correlation calculated by Spearman‘s rank correlation coefficient. *p<0.05,**p<0.01,***P<0.001.

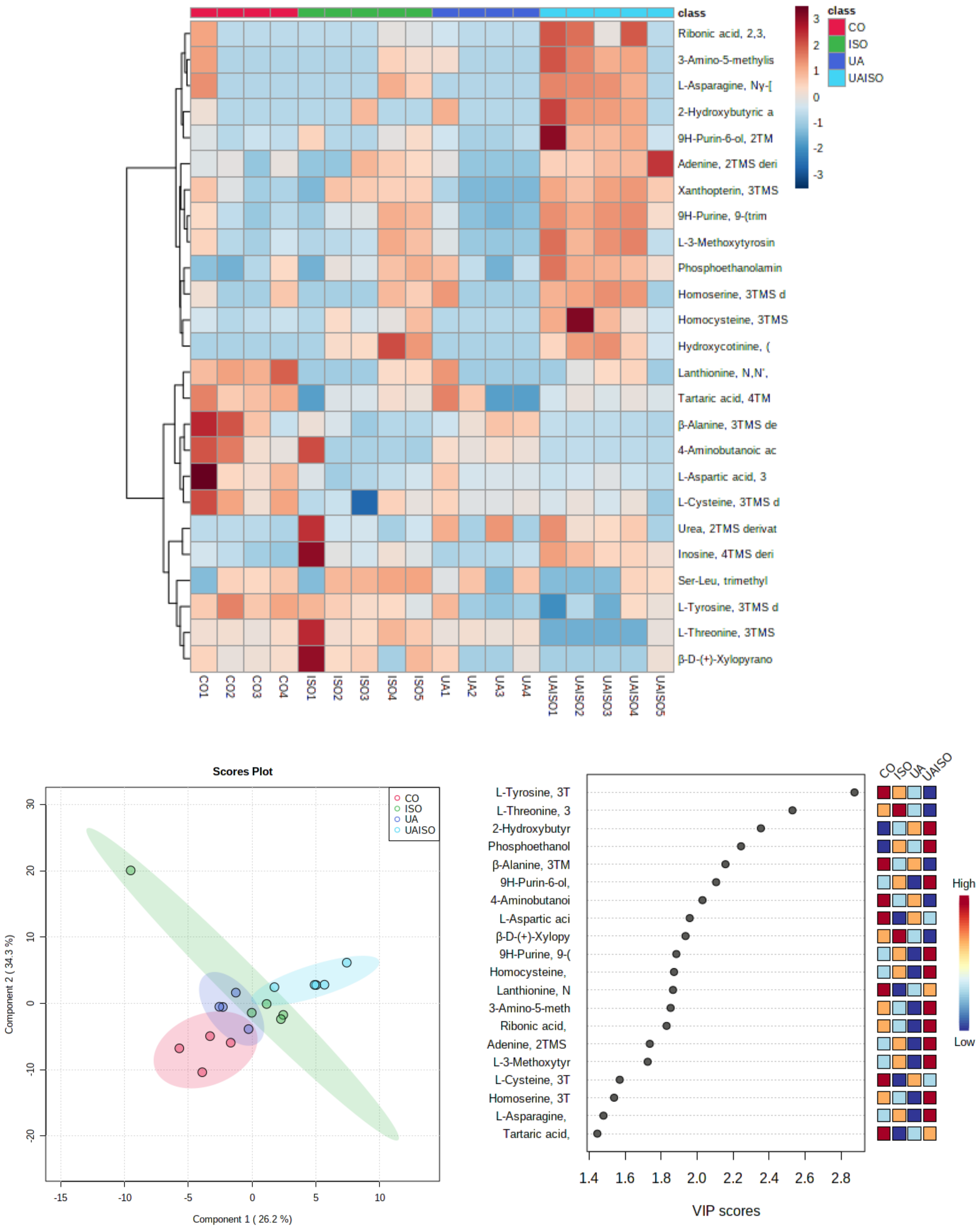

Figure S2. UA aggravates ISO induced heart failure leads to impaired energy metabolism in zebrafish.

A. Heatmap of several metabolites in UA, ISO and co-treatment induced heart failure zebrafish larvae at 96 hpf. The color in each cell represents the expression of each zebrafish cluster sample as the scale bar showed, each zebrafish cluster includes 50 zebrafish larvae. B PCA analysis of every sample among each group. C. VIP scores of the top metabolites among each group.

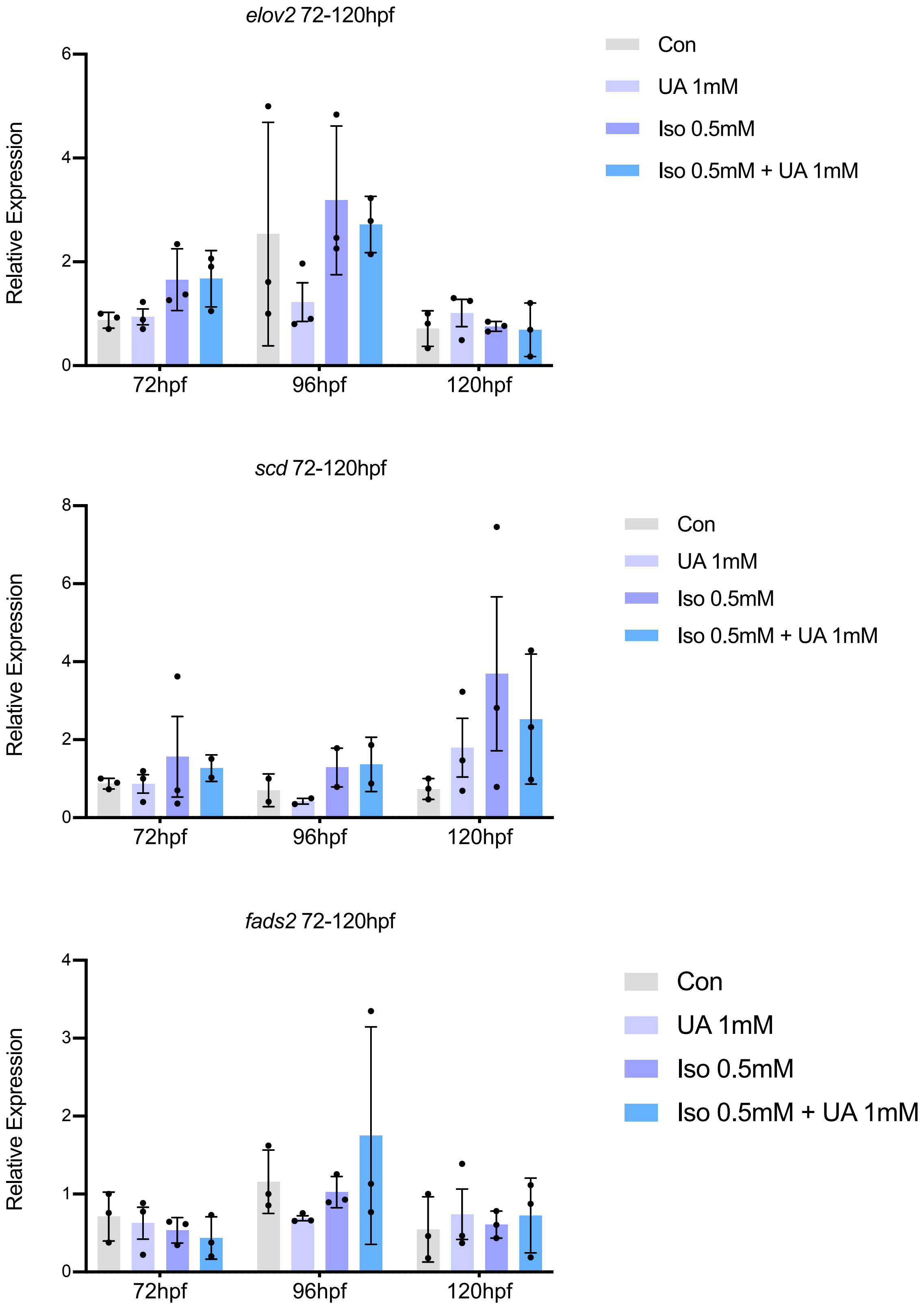

Figure S3. Expression analyses of elov2,scd and fads2 in zebrafish after UA and ISO treatment.
1mM UA treatment and 0.5mM ISO induced elov2,scd and fads2 mRNA expression in *Tg(fli1:EGFP)* zebrafish larvae at 72, 96 and 120 hpf. Expression of mRNA was analysed by RT-qPCR and was normalized to both *b-actin* and *b2m*, each single dot represents one zebrafish cluster's data, which includes 20-30 zebrafish larvae.For statistical analysis one-way ANOVA followed by Sidak's multiple comparison test was applied, *p < 0.05, **p < 0.01, ***p<0.001, ****p < 0.0001. Fads2, fatty acid desaturase 2; elov2. fatty acid elongase 2; scd, stearoyl-CoA desaturase.

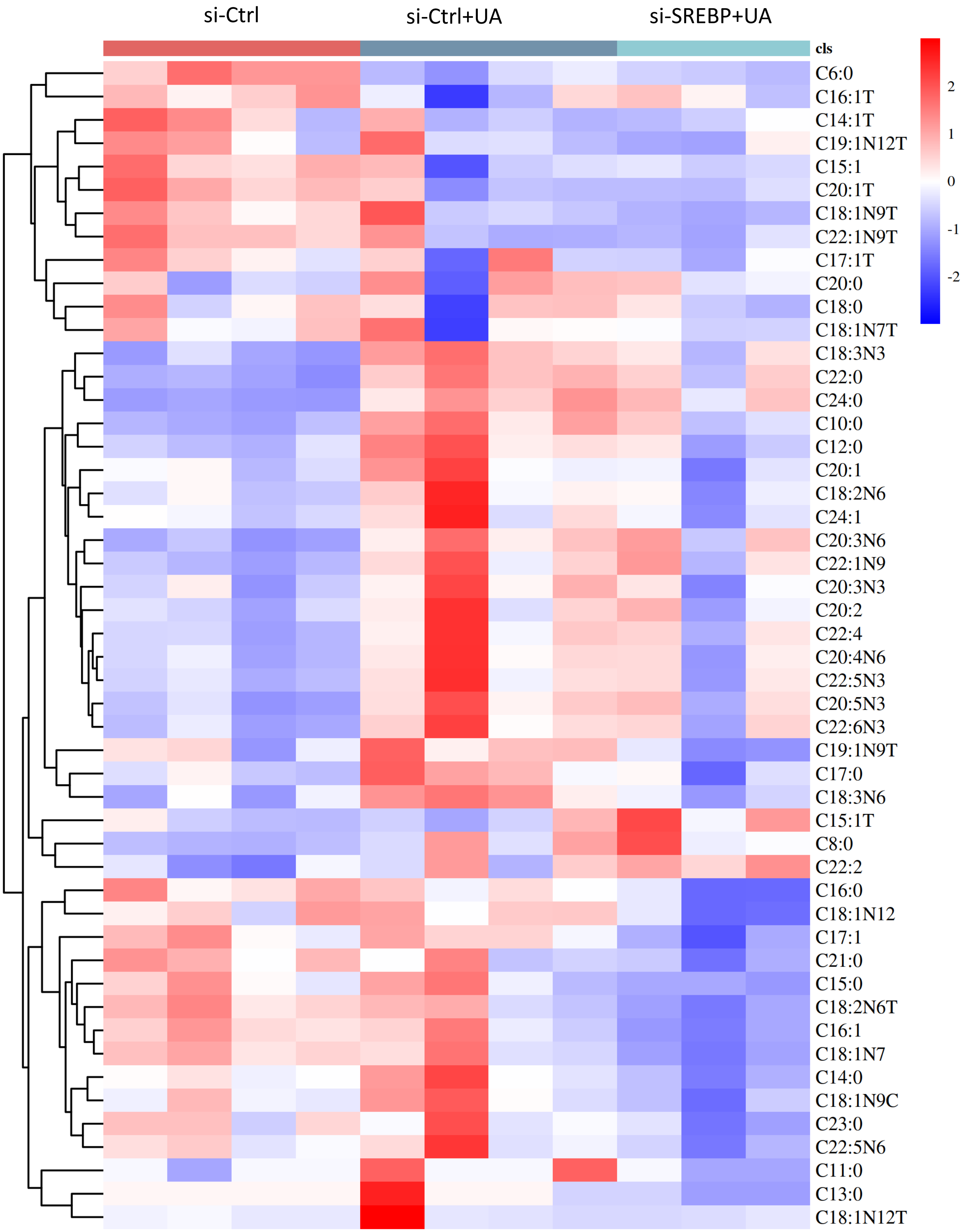

Figure S4. Heatmap of fatty acids in HepG2 after UA treatment and Srebp1 knockdown.
Heatmap of fatty acids in HepG2 after UA treatment and Srebp1 knockdown. The color in each cell represents expression of each cell culture sample as the scale bar showed, n = 3-4 per group.

qPCR primer name	Primer sequence
For zebrafish	
β-actin-qPCR-for	ACGGTCAGGTCATCACCATC
β-actin-qPCR-rev	TGGATACCGCAAGATTCCAT
b2m-qPCR-for	ACTGCTGAAGAACGGACAGG
b2m-qPCR-rev	GCAACGCTCTTTGTGAGGTG
nppb-qPCR-for	TCCCGTAGTCGGCCTTCTCT
nppb-qPCR-rev	TCCGCCGGTGTTTGGTCTTG
elovl2-qPCR-for	TGGACAGCCTATTTGGAGAAA
elovl2-qPCR-rev	AATGTTGGTGTGTAGGAATCCA
fads2-qPCR-for	CGTCGCTGTTATTCTGGCTA
fads2-qPCR-rev	ACGGACAGATGACCGAAGTC
Scd-qPCR-for	GGTCCACGTGTTTAGAGCAGT
Scd-qPCR-rev	GGGTCAAACTCATCCTCCATT
Fasn-qPCR-for	AGTGTGCCGTGCTATGGACT
Fasn-qPCR-rev	CGCAGCAAGACTCTGGATACT
For Cell Culture	
β-actin-qPCR-for	CATGTACGTTGCTATCCAGGC
β-actin-qPCR-rev	CTCCTTAATGTCACGCACGAT
Fasn-qPCR-for	CAACCTCTCCCAGGTATGCG
Fasn-qPCR-rev	CCAGGGAGCTGTGGATGATG
Srebp1-qPCR-for	GCTCCCTAGGAAGGGCCGTA
Srebp1-qPCR-rev	GCTCCCTAGGAAGGGCCGTA

Table.S1 Primers.

	B	β	t	p	F	Adjusted R^2
**Age**	-0.160	-0.229	-1.933	0.057	2.077*	0.09
**Sex**	-3.027	-0.194	-1.700	0.093		
**UA(umol/L)**	-0.026	-0.297	-2.052	0.043		
**AST(U/L)**	0.064	0.092	0.692	0.491		
**ALT(U/L)**	-0.032	-0.045	-0.326	0.745		
**Cre(μmol/L)**	0.021	0.064	0.432	0.667		
**LDL-C(mmol/L)**	-0.282	-0.033	-0.293	0.770		
**HbA1C(%)**	-0.278	-0.047	-0.434	0.666		

Table.S2 Multiple linear regression analysis in the metabolic syndrome cohort (big cohort).
Dependent variable: EF value. UA, uric acid; ALT, alanine transaminase; AST, aspartate transaminase; Cre, creatinine; LDL-C, high density lipoprotein; *p<0.05.

	Normal EF	HErEF	P Value
**Age**	58.22±12.47	64.16±9.92	ns
**Sex(Female%)**	43	0.42	ns
**EF(%)**	67.91±5.37	41.22±6.46	P<0.001
**LVEDD(mm)**	49.00±3.64	63.27±11.7	P<0.001
**LVESD(mm)**	29.68±3.30	50.49±12.13	P<0.001
**UA(umol/L)**	276.72±82.43	366.90±92.00	P<0.001
**AST(U/L)**	21.41±5.42	51.97±5.03	P<0.001
**ALT(U/L)**	20.68±13.44	37.35±17.05	P<0.001
**Cre(μmol/L)**	61.48±15.08	96.52±38.21	0.22
**Chol(mmol/L)**	4.11±1.29	3.93±1.01	ns
**TG(mmol/L)**	1.50±0.81	1.57±0.73	ns
**HDL-C(mmol/L)**	1.11±0.39	0.94±0.21	ns
**LDL-C(mmol/L)**	2.34±0.95	2.40±0.94	ns
**HbA1C(%)**	5.61±0.46	5.91±0.69	P<0.001

Table.S3 Baseline information of the heart failure cohort (small cohort for metabolomics detection)
EF, ejection fraction; LVEDD, left ventricular end diastolic diameter; LVESD, left ventricular end systolic diameter; UA, uric acid; ALT, alanine transaminase; AST, aspartate transaminase; Cre, creatinine; Chol, cholesterol;TG, triglycerides.HDL-C, high density lipoprotein; LDL-C, high density lipoprotein;
